# Human Folliculin Delays Cell Cycle Progression through Late S and G2/M-Phases: Effect of Phosphorylation and Tumor Associated Mutations

**DOI:** 10.1371/journal.pone.0066775

**Published:** 2013-07-11

**Authors:** Laura A. Laviolette, Jonas Wilson, Julia Koller, Christopher Neil, Peter Hulick, Tomas Rejtar, Barry Karger, Bin Tean Teh, Othon Iliopoulos

**Affiliations:** 1 Center for Cancer Research at The Massachusetts General Hospital Cancer Center, Charlestown, Massachusetts, United States of America; 2 Division of Hematology-Oncology, Department of Medicine, Massachusetts General Hospital Cancer Center, Boston, Massachusetts, United States of America; 3 Department of Medicine, Harvard Medical School, Boston, Massachusetts, United States of America; 4 Department of Chemistry and Chemical Biology and the Barnett Institute, Northeastern University, Boston, Massachusetts, United States of America; 5 Laboratory of Cancer Genetics, Van Andel Research Institute, Grand Rapids, Michigan, United States of America; 6 NCCS-VARI Translational Research Laboratory, National Cancer Centre Singapore, Singapore; Rush University Medical Center, United States of America

## Abstract

The Birt-Hogg-Dube disease occurs as a result of germline mutations in the human Folliculin gene (*FLCN*), and is characterized by clinical features including fibrofolliculomas, lung cysts and multifocal renal neoplasia. Clinical and genetic evidence suggest that *FLCN* acts as a tumor suppressor gene. The human cell line UOK257, derived from the renal cell carcinoma of a patient with a germline mutation in the *FLCN* gene, harbors a truncated version of the FLCN protein. Reconstitution of the wild type FLCN protein into UOK257 cells delays cell cycle progression, due to a slower progression through the late S and G2/M-phases. Similarly, *Flcn* ^–/–^ mouse embryonic fibroblasts progress more rapidly through the cell cycle than wild type controls (*Flcn* ^flox/flox^). The reintroduction of tumor-associated FLCN mutants (FLCN ΔF157, FLCN 1–469 or FLCN K508R) fails to delay cell cycle progression in UOK257 cells. Additionally, FLCN phosphorylation (on Serines 62 and 73) fluctuates throughout the cell cycle and peaks during the G2/M phase in cells treated with nocodazole. In keeping with this observation, the reintroduction of a FLCN phosphomimetic mutant into the UOK257 cell line results in faster progression through the cell cycle compared to those expressing the wild type FLCN protein. These findings suggest that the tumor suppression function of FLCN may be linked to its impact on the cell cycle and that FLCN phosphorylation is important for this activity. Additionally, these observations describe a novel *in vitro* assay for testing the functional significance of FLCN mutations and/or genetic polymorphisms.

## Introduction

Individuals with a germline mutation in the *FLCN* gene have a high lifetime risk for developing multiple and bilateral renal cell carcinomas of any histologic type, fibrofolliculomas of the skin, and lung cysts that can lead to spontaneous pneumothorax. This constellation of lesions is known as Birt-Hogg-Dube (BHD) syndrome [1], [2], [3], [4], [5], [6], [7].

The gene responsible for the BHD syndrome, *FLCN*, was identified by linkage analysis and recombination mapping in BHD families [8]. Clinical and genetic evidence indicate that *FLCN* acts as a tumor suppressor gene. Analysis of renal cell carcinomas from BHD patients reveals either loss of the wild type allele or a second, somatic mutation that inactivates the wild type allele [9], [10]. Deletion of the *Flcn* gene in the kidneys of transgenic mice leads to the development of renal cysts and renal cell carcinoma [11], [12], [13]. In addition, the reintroduction of the wild type (WT) FLCN protein into the FLCN-deficient human renal cell carcinoma cell line UOK257 suppresses their growth as colonies in soft agar and restricts their growth as tumors when xenografted in SCID mice [14], [15].

*FLCN* consists of 14 exons and encodes an evolutionarily conserved, nuclear and cytoplasmic, 64 kDa phosphoprotein, folliculin (FLCN), which is ubiquitously expressed in adult and embryonic tissues and has no obvious functional domains [8], [16], [17], [18]. Little is known about the biochemical function(s) of the FLCN tumor suppressor protein. Cytoplasmic FLCN interacts with Folliculin Interacting Proteins 1 and 2 (FNIP1 and FNIP2) in a phosphorylation-dependent manner, and together they enter complexes containing AMPK [16], [19], [20]. The functional outcome of this biochemical interaction and the mechanistic details of FLCN-FNIP-AMPK signaling remain unclear. Opposing data have been provided indicating that FLCN down-regulates [11], [16] or up-regulates [13], [21] mTORC1 function *in vitro* and *in vivo*.

To understand how FLCN may restrict cell growth *in vitro*, we investigated its effect on the cell cycle. We synchronized isogenic derivatives of the human renal cell carcinoma cell line UOK257 that express either WT FLCN, mutant FLCN, or the vector (control) and we studied their progression through the cell cycle. Here we provide evidence that WT, but not tumor-associated FLCN mutants, delay cell cycle progression through the late S and G2/M phase. Similarly, mouse embryonic fibroblasts (MEFs) lacking the *Flcn* gene progressed more rapidly through the cell cycle than control MEFs expressing WT *Flcn*. During these studies we observed that FLCN phosphorylation fluctuates during the cell cycle and that FLCN phosphorylation on serines 62 (S62) and 73 (S73) increases dramatically at the G2/M boundary. We further showed that orderly phosphorylation of FLCN is required for its effect on late S and G2/M progression. This paper describes a fast assay for analyzing the function of the FLCN protein and it links FLCN phosphorylation to cell cycle progression. Thus it provides an investigational frame to further study the role of FLCN on cell cycle regulation.

## Materials and Methods

### Cell lines

The UOK257 renal carcinoma cell line (a generous gift from Drs. Marston Linehan and Laura Schmidt, NCI/NIH) was originally derived from the clear cell renal tumor of a BHD patient and was previously established and described [15], [22]. The cells were grown in Dulbecco's Modified Eagle Medium (DMEM, Invitrogen, Carlsbad, CA) supplemented with 10% fetal calf serum (Hyclone), penicillin, streptomycin and L-glutamine (Invitrogen, Carlsbad, CA). Mycoplasma testing was performed regularly to ensure that the cells were mycoplasma negative. The UOK257 cells were infected with retroviruses encoding for FLCN or FLCN mutants and selected in 2.0 micrograms/ml of puromycin. MEF cells were obtained from a genetically engineered conditional *Flcn* knockout mouse, *BHD* ^flox/flox^ [12]. All mice were housed and manipulated in an ethically and humane manner, according to protocols approved by the Institutional Animal Care and Use Committee (IACUC) of Van Andel Institute. The MEF cells were spontaneously immortalized by serially passaging them, according to the NIH 3T3 protocol [23], [24], in DMEM GlutaMAX media (Invitrogen) supplemented with 10% fetal bovine serum (Hyclone), penicillin, streptomycin and L-glutamine (Invitrogen). The cells were infected *in vitro* with adenovirus expressing Cre recombinase and clones were screened for recombination as previously described [12]. Cellular clones exhibiting 100% recombination in the *Flcn* gene were used in cell cycle experiments and mock infected clones were used as controls.

### Plasmids

The human WT FLCN gene was generated from a HEK293 cDNA pool by PCR with oligonucleotides 5′-GCGCGGATCCGCCACCATGAATGCCATCGTGGCTCTCTG-3′ (forward) and 5′-GCGCGAATTCAGTTCCGAGACTCCGAGGCTGTG-3′ (reverse). The PCR product was restricted with BamHI and EcoRI and ligated into pBABE-puromycin vector plasmid. Several mutant forms of FLCN were generated, including two phosphomutants (phosphomimetic and phosphoinactivating) and three tumor-associated mutants (FLCN 1–469, FLCN K508R, and FLCN ΔF157). For the FLCN 1–469 mutant the reverse oligonucleotide 5′-GCGCGAATTCAACTGGTCACCACAAACTCGTACT TG-3′ was used. FLCN K508R mutant was engineered by PCR mutagenesis of the wild type FLCN using oligonucleotides 5′-TCGTCTGCCTCAGGGAGGAGTGG-3′ (forward) and 5′-CCACTCCTCCCTGAGGCAGACGA-3′ (reverse). FLCN ΔF157 mutant was engineered by PCR mutagenesis of the wild type FLCN using oligonucleotides 5′-AGCCACACCTTCATCAAGGACAGC-3′ (forward) and 5′-GCTGTCCTTGATGAAGGTGTGGCT -3′ (reverse). The FLCN S62A/S73A and FLCN S62E/S73E phosphomutants were generated using the following oligonucleotides: S62A, 5′-CGTGCGCACGCCCCCGCAGAG-3′ and 5′-CTCTGCGGGGGCGTGCGC ACG-3′; S62E, 5′-CGTGCGCACGAGCCCGCAGAG-3′ and 5′-CTCTGCGGGCTCGT GCGCACG-3′; S73A, 5′-GAGTCCAGCGCCCCGGGGCCC-3′ and 5′-GGGCCCCGGGGCGCTGGACTC-3′; S73E, 5′-GAGTCCAGCGAGCCGGGGCCC-3′ and 5′-GGGCCCCGGCTCGCTGGACTC-3′. Retroviruses were produced by co-transfecting 293T cells as described previously [25]. The media containing the virus was collected, filtered and the UOK257 cells were infected by spin infection [26].

### Western blots and antibodies

Protein expression was detected by Western blotting, as described before [25]. In brief, for cell lysis, RIPA buffer (25 mM Tris•HCl pH 7.6, 150 mM NaCl, 1% NP-40, 1% sodium deoxycholate, 0.1% SDS) containing protease and phosphatase inhibitors (Trypsin Inhibitor, Leupeptin, Sodium Orthovanadate, Pepstatin A, Aprotinin, Sodium Fluoride, and PMSF) was used. Proteins were separated by SDS-PAGE electrophoresis, transferred to a PVDF membrane and detected with the cognate antibody. The following antibodies were used: anti-Pan Actin (Neomarkers, Fremont, CA); anti-FLCN antibody (FLCN (D14G9) Rabbit mAb #3697; Cell Signaling Technology, Danvers, MA). The antigen recognized by the anti-human FLCN antibody maps to amino acids 301–430 (data not shown). For protein stability experiments, cells were treated with 10 μg/ml of cycloheximide and collected over the course of 8 hours.

### Cell fractionation

The UOK257 cells were gently lysed in a hypotonic solution on ice for 15 minutes. The nuclei were pelleted (30 sec at 18400×g) and the supernatant containing the cytosolic fraction was collected. The nuclear fraction was isolated by resuspending the remaining cell pellet in a high salt solution (20 mM Tris-HCl pH 7.5, 100 mM KCl, 2 mM MgCl_2_, 1 mM CaCl_2_, 300 mM sucrose, and 0.1% Triton X-100) and rotating for 15 minutes at 4°C. The solution was centrifuged at 18400×g for 5 minutes and the supernatant containing the nuclear fraction was collected.

### Post-translational modifications detection by LC-MS/MS

*In gel digestion*. FLCN was immunoprecipitated from cells arrested in G2/M by nocodazole (50 ng/ml for 14–16 hours) and resolved in SDS-PAGE. Gels were stained with Coomassie Blue and FLCN bands subjected to in-gel tryptic digestion for 18 hours at 37°C. *Liquid Chromatography – Mass Spectrometry*. The protein digests were analyzed by capillary reversed phase liquid chromatography (RPLC), on-line coupled to a hybrid LTQ−FT MS (Thermo Fisher Scientific, San Jose, CA). The nano-RPLC system was composed of an Ultimate 3000 nano−LC pump (Dionex, Sunnyvale, CA), a 75 µm i.d. 15 cm analytical column packed with 3 µm i.d. Magic C18AQ, 200 Å pore size particles, with a flow rate of 200 nl/minute. MS data were collected in the data dependent mode. MS scans were acquired from *m/z* 400 to 2000 with a mass resolution of 100,000 at *m/z* 400, with up to 2×10^6^ ions and a normalized collision energy of 35%. *Data Processing*: The DTA files were generated from acquired raw files by ExtractMSn program (Version 4.0, Thermo Fisher Scientific), converted to Mascot generic format and searched against the human SwissProt database using the Mascot algorithm (ver. 2.2, MatrixScience, Boston, MA). Cysteine carbamidomethylation was included as a fixed modification while phosphorylation of S, T, or Y was considered a variable modification. Full tryptic enzyme specificity was applied with up to two missed cleavage sites. Precursor mass tolerance was set to 30 ppm with correction for up to two ^13^C isotopes to account for incorrectly assigned monoisotopic peaks, and 0.7 Da was the tolerance for the fragment ions. MS/MS spectra assigned to human FLCN peptides with phosphorylation were inspected manually to determine the exact site of modification.

### Cell synchronization and cell cycle progression analysis

Cells were synchronized using nocodazole or a double thymidine block. Sub-confluent cells were treated with nocodazole (50 ng/ml) for 14–16 hours and only the mitotic cells were collected. For cell cycle progression analysis, cells were grown to about 20–30% confluence in 6 well dishes. Cells were arrested at G1/S by treatment with 2 mM thymidine for 18 hours. The cells were then released by washing with PBS and grown in DMEM for 9 hours. For the second block, cells were treated with thymidine (2 mM) for 17 hours. Double blocked cells were released by washing with PBS and adding fresh medium. Cell cycle progression was measured using the Click-it EdU Flow Cytometry Assay Kit (used according to manufacturer's instructions, Invitrogen). The samples were processed and analyzed using a BD LSR II Flow Cytometer System and BD FACSDiva software (BD Biosciences, San Jose, CA).

## Results

**Figure 1:**
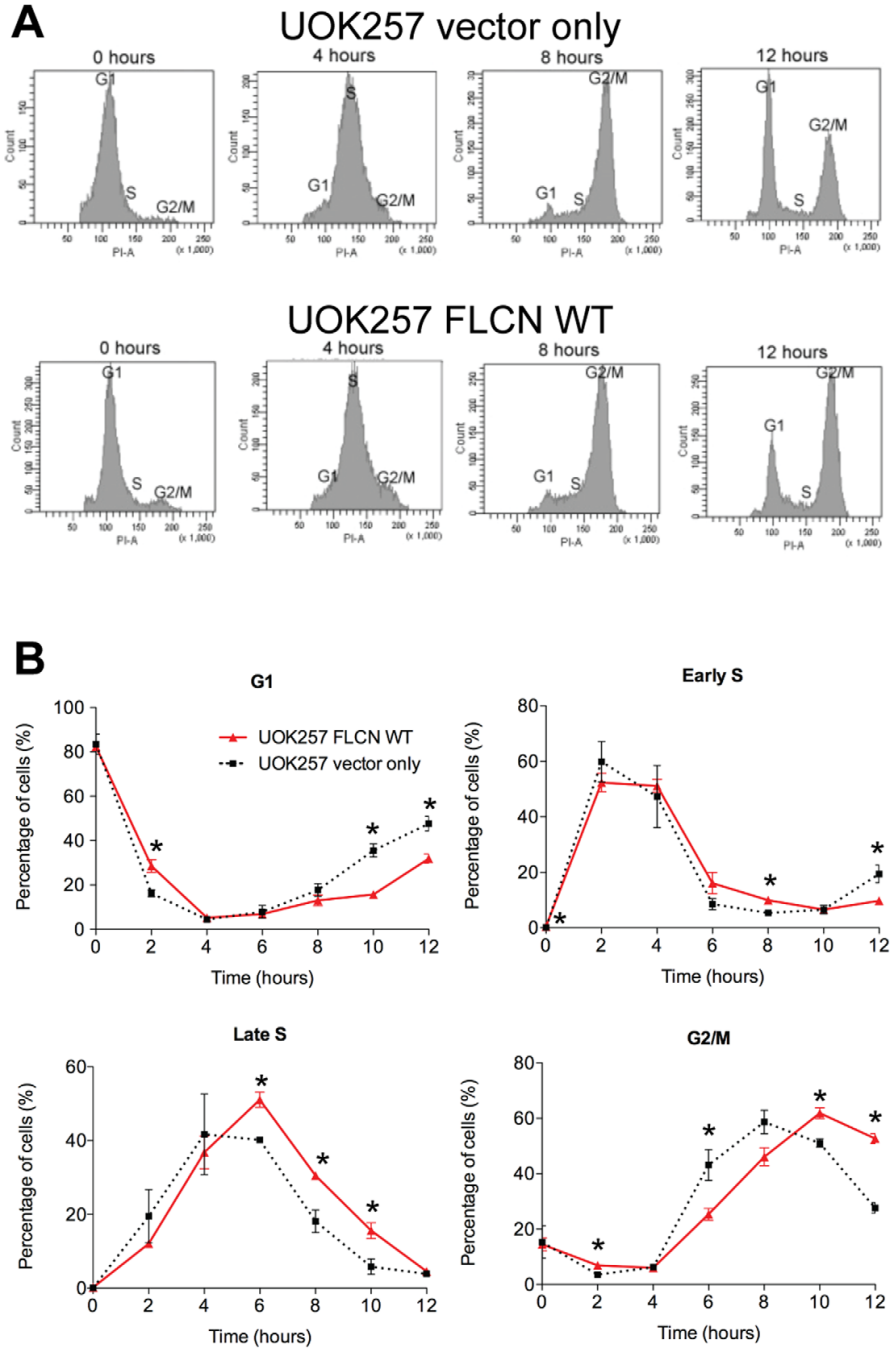
FLCN delays progression through the late S and the G2/M phases of the cell cycle. (A) Representative cell cycle profiles of UOK257 cells infected with retroviruses expressing the vector only or WT FLCN. Progression through the cell cycle was measured by propidium iodide (PI) labeling of synchronized cells collected at 0, 4, 8 and 12 hours after release from thymidine block. (B) FLCN-deficient UOK257 cells (squares and dashed line) and their isogenic counterparts reconstituted with wild type (WT) FLCN (triangles and solid line) were synchronized by double thymidine block and their progression through cell cycle after release was analyzed by FACS. Vertical axis indicates the percent of all cells registering in that specific phase of cell cycle. Horizontal axis indicates hours post release from thymidine. The average of three experiments is presented (n = 3), * indicates a statistically significant difference (t test, p<0.05), bars correspond to standard error of the mean (SEM).

Genetic evidence suggests that FLCN acts as a tumor suppressor gene. Since several tumor suppressor genes affect the cell cycle, we sought to investigate the potential role of FLCN on cell cycle progression. In order to investigate the cellular functions of FLCN, we used the human cell line UOK257, which was generated from the renal cell carcinoma of a BHD patient and expresses a truncated, putatively null form of the FLCN protein. The UOK257 cells were infected with retroviruses that express WT human FLCN or the empty vector (control; FLCN null). The effect of FLCN reconstitution on cell cycle progression was determined by comparing the cell cycle profiles (both propidium iodide staining (Figure 1A) and EdU uptake (Figure 1B)) of these two isogenic cell lines following synchronization with a double thymidine block. FLCN WT reconstitution resulted in a statistically significant delay in progression through the late S phase (6–8 hours after release from thymidine treatment), which continued during the G2 and M phases (8–12 hours) of the cell cycle (Figure 1A and 1B). The FLCN null cells (UOK257 vector only) progressed more rapidly through the cell cycle (late S phase at 4–6 hours and G2/M phases at 6–10 hours after release from thymidine; Figures 1A and 1B). There was no difference in the response of FLCN deficient or FLCN WT reconstituted cells to thymidine block and both populations arrested similarly in G1 (Figure 1B, 0 hour time point).

**Figure 2:**
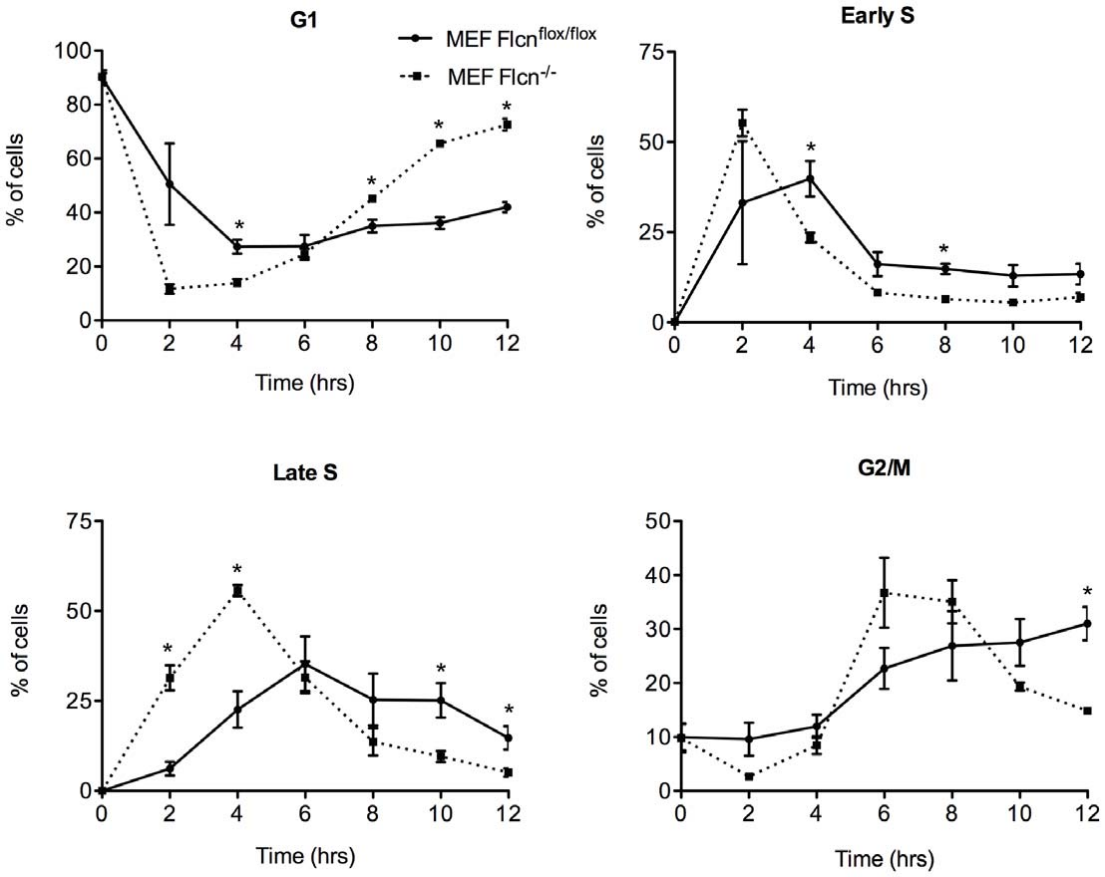
MEF cells lacking the *Flcn* gene progress more rapidly through the cell cycle. MEF cells with a floxed copy of the *Flcn* gene (*Flcn* ^flox/flox^; circles and solid line) and MEF cells null for *Flcn* following Cre recombinase mediated excision and recombination (*Flcn* ^–/–^; squares and dashed line) were synchronized by double thymidine block. FACS analysis was used to determine the percentage of cells in each stage of the cell cycle (y axis) over the course of 12 hours (x axis). Error bars indicate SEM from three experiments (n = 3), * indicates a statistically significant difference (t test, p<0.05).

To confirm these results in a second, non-transformed cell line, we immortalized MEF cells homozygous for a floxed copy of the *Flcn* gene (*Flcn* ^flox/flox^). *Flcn* knockout cells (*Flcn* ^–/–^) were obtained by infecting the *Flcn* ^flox/flox^ MEFs *in vitro* with adenoviruses expressing Cre recombinase. MEF cells lacking the *Flcn* tumor suppressor gene (*Flcn* ^–/–^) progressed more rapidly through G1, early S, late S and G2/M than *Flcn^flox/flox^* MEFs (Figure 2).

**Figure 3:**
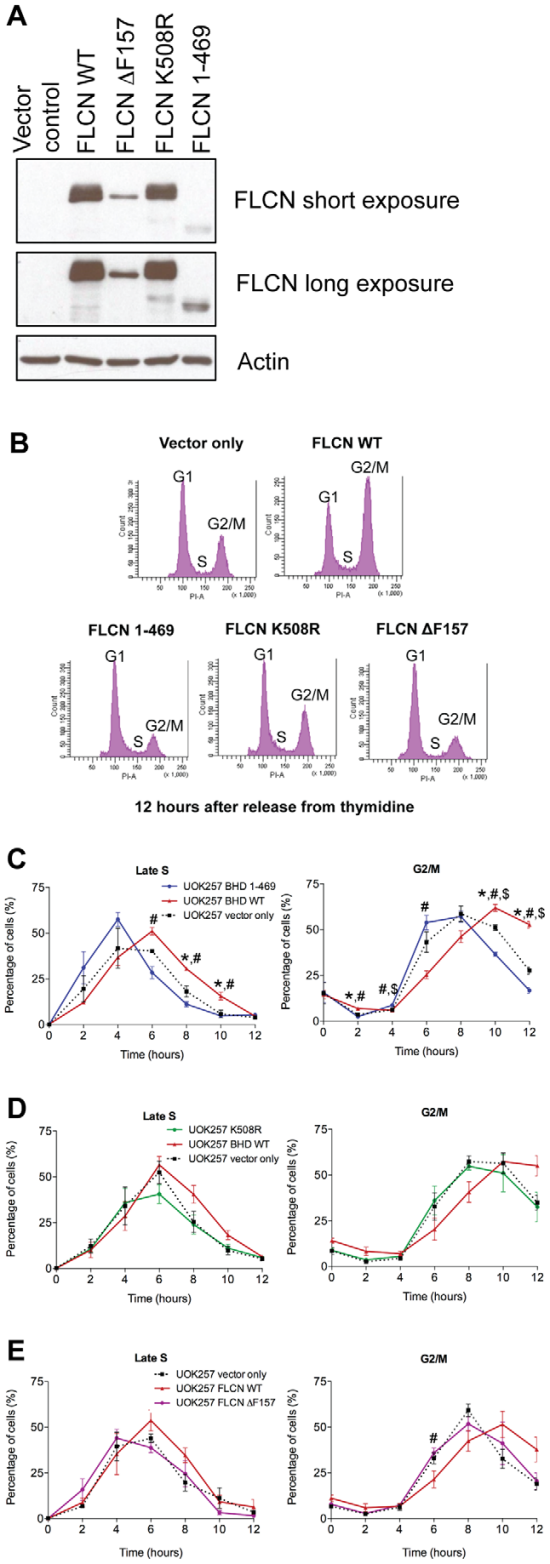
Tumor-associated *FLCN* mutations fail to delay cell cycle progression. (A) Expression of exogenous FLCN in UOK257 cells infected with retroviruses harboring empty vector control, FLCN WT, FLCN ?F157, FLCN K508R, or FLCN 1–469. (B) Cell cycle profiles of the UOK257 cells and the isogenic cell lines expressing empty vector control, FLCN WT, FLCN ΔF157, FLCN K508R, or FLCN 1–469, demonstrating the amount of cells in the G1, S and G2/M stages as determined by PI staining. The experiment was repeated three times (n = 3), but only one representative image of the cell cycle profile, 12 hours after release from thymidine block, is shown. (C) *Tumor-associated FLCN truncated mutant 1–469 fails to delay cell cycle progression*. FLCN deficient UOK257 cells (black dashed line) and their isogenic counterpart reconstituted with FLCN WT (red line) or FLCN 1–469 mutant (blue line) were synchronized by double thymidine block and released. Progression through the cell cycle was analyzed by FACS. Vertical axis indicates the percentage of all cells registering in the specific phase of cell cycle. Horizontal axis indicates hours post thymidine release. (D) *Tumor-associated FLCN missense mutant K508R fails to delay cell cycle progression*. Same as in (C), except that the reconstituted mutant is FLCN K508R (green line). (E) *Tumor-associated FLCN deletion mutant ΔF157 fails to delay cell cycle progression*. Same as in (C) and (D), except that the reconstituted mutant is FLCN ?F157 (purple line). The average of three experiments is presented (n = 3) in each panel; * indicates a significant difference between UOK257+ vector only compared to UOK257+ *FLCN* WT; # indicates a difference between UOK257 *FLCN* WT and the UOK257+ tumor-associated mutant (1–469, K508R, or ?F157); $ indicates a difference between UOK257+ vector only cells and the UOK257 cells expressing a tumor-associated mutant (One way ANOVA, Tukey's post-test, p<0.05). Error bars correspond to SEM.

To investigate whether FLCN's effect on cell cycle progression is linked to its tumor suppressor activity, the FLCN null UOK257 cells were reconstituted with either a tumor-associated FLCN missense point mutant (K508R), a tumor-associated FLCN truncation mutant (amino acids 1–469), or a tumor-associated FLCN in-frame deletion (ΔF157). These mutations have been detected in the germline of BHD patients and have been linked to the development of fibrofolliculomas, pneumothorax and renal cell carcinoma [10], [27], [28]. FLCN K508R is expressed at levels comparable to the exogenously expressed WT FLCN, while the truncating mutants FLCN 1–469 and FLCN ΔF157 display decreased stability (Figure 3A), which is in agreement with previously reported data demonstrating reduced protein stability for tumor-associated FLCN truncating mutants [29]. All of the tumor-associated mutants fail to delay cell cycle progression through the S and G2/M phases (Figure 3B–E), suggesting a genetic link between FLCN's tumor suppressor function and its ability to control cell cycle progression.

**Figure 4:**
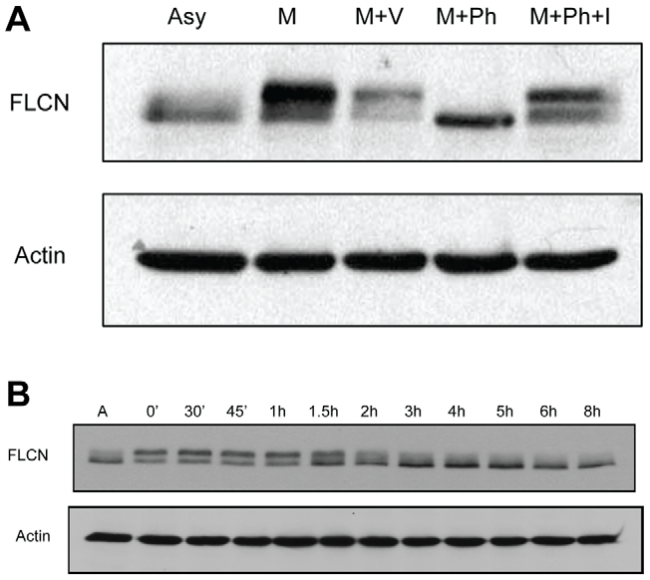
FLCN is hyperphosphorylated during cell cycle progression from G2 to M. (A) *A slower migrating FLCN band is detected in cells arrested at the G2/M boundary and is likely the result of phosphorylation*. The expression of endogenous human FLCN in nocodazole arrested, mitotic (M) U20S cells was analyzed by FLCN immunoblot after a 2 hour incubation with vehicle only (M+V), phosphatase (M+ Ph), or in the presence of phosphatase along with phosphatase inhibitors (M+ Ph +I). U20S cells growing asynchronously (Asy) were used as a control (hypophosphorylated). (B) *FLCN phosphorylation decreases upon exit from mitosis*. U2OS cells arrested by nocodazole in G2/M were collected by mitotic shake and replated. FLCN expression was detected by Western blot at increasing time intervals post-replating (time indicated on the horizontal axis). Actin is used as a loading control.

**Figure 5:**
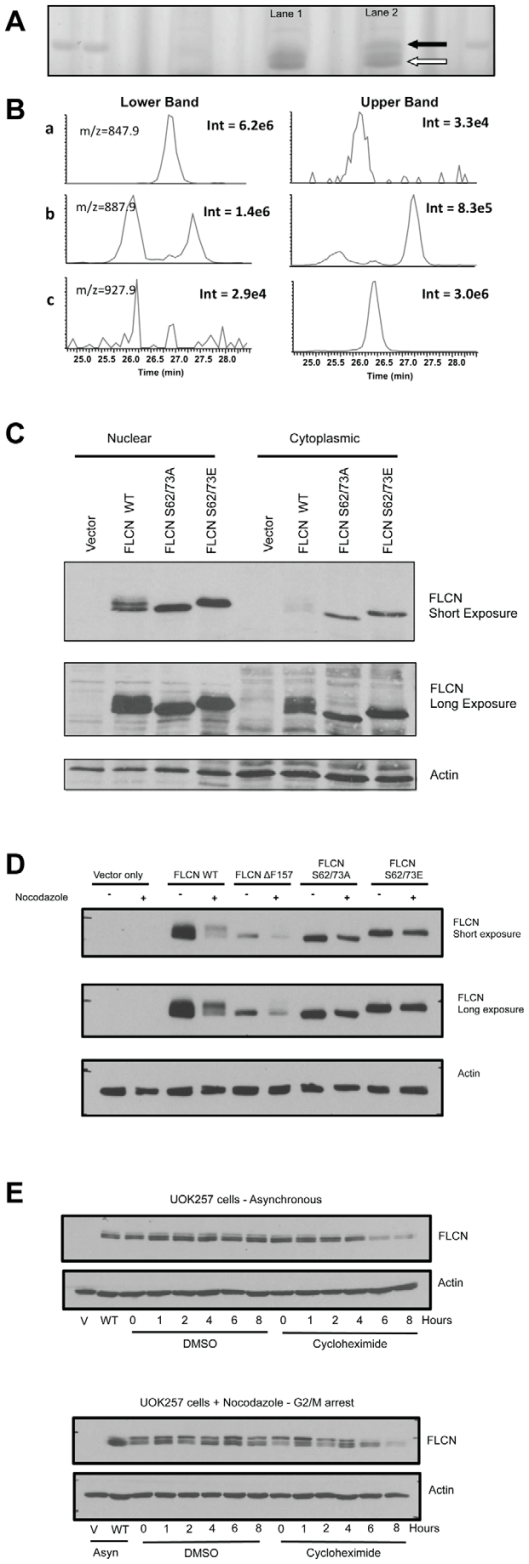
Identification of FLCN phosphorylation sites at the G2/M boundary. (A) U2OS cells were cultured in the presence (lane 2) or absence (lane 1) of nocodazole overnight and immunoprecipitated FLCN protein was detected by Coomassie Blue staining of SDS-PAGE gels. (B) LC-MS/MS analysis of FLCN peptides. Extracted ion chromatograms for FLCN peptide AHSPAEGASVESSSPGPK with no phosphorylation (a), one phosphorylation (b) and 2 phosphorylation sites (c). Intensity corresponds to the absolute intensity of the highest peak in the chromatogram. No normalization was performed. (C) FLCN deficient UOK257 cells were reconstituted with FLCN WT, FLCN double phosphoinactivating mutant S62/73A or FLCN double phosphomimetic mutant S62/73E. Exogenous WT FLCN and the phosphomutants (S62/73A and S62/73E) are expressed in both the nuclear and the cytoplasmic cellular fractions. (D) FLCN deficient UOK257 cells and the isogenic cell lines reconstituted with either FLCN WT, a tumor-associated mutant (FLCN ?F157) or the phosphomutants (S62/73A and S62/73E) were cultured overnight in the absence or presence of nocodazole. (E) UOK257 FLCN WT cells growing asynchronously (Asyn) or arrested during G2/M with nocodazole were treated with either DMSO or cycloheximide (10 μg/ml). Protein stability was determined by Western blot at various time points over the course of 8 hours (V – UOK257 vector only cells; WT – UOK257 FLCN WT cells). Lysates were resolved in SDS-PAGE and FLCN protein detected with anti-FLCN antibody. Actin serves as a loading control.

Following our observation that the loss of WT FLCN results in faster progression through the cell cycle, we examined changes in the level of FLCN protein expression during the cell cycle. No significant changes in FLCN expression were detected during the first 8 hours after release from the double thymidine block (G1 through to late S phase), but the formation of slow migrating FLCN species was detected at the time corresponding to late G2 phase (data not shown). To further investigate changes in endogenous FLCN expression during the late G2 phase, we arrested U2OS osteosarcoma cells at the G2/M boundary with nocodazole. U20S cells endogenously express WT FLCN, which appears as a doublet by Western blot. During mitosis (immediately following nocodazole treatment), there was a shift in the Western blot banding pattern, and the FLCN protein was mainly detected in the top band of the doublet, which migrates with slower mobility (Figure 4A). This slow migration is likely due to phosphorylation of FLCN since treatment of the mitotic cell protein lysates with phosphatase attenuates the expression of this slower FLCN species (Figure 4A). To determine if FLCN phosphorylation is altered during the cell cycle, especially during the G2 and M phases, we replated the mitotic cells and examined FLCN expression at specific time intervals after mitosis. The Western blot data demonstrated that endogenous FLCN phosphorylation changed over time and returned to baseline following exit from mitosis, which occurs in about 1.5–2 hours after release from nocodazole (Figure 4B). Enhancement of FLCN phosphorylation at the G2/M boundary is not a cell type specific phenomenon; it has been detected in the U20S, HeLa and UOK257 cell lines (Figure 5D, and data not shown).

To identify the residues that are phosphorylated at the G2/M boundary, we immunoprecipitated endogenous FLCN from asynchronous or nocodazole arrested U2OS cells. The precipitates were resolved in SDS-PAGE gels and the FLCN protein was detected by size using Coomassie Blue staining (Figure 5A). The lower (Figure 5A, open arrow) and the upper (Figure 5A, filled arrow) migrating FLCN bands were excised separately and in gel digested with trypsin. The post-translational modifications of the peptides were analyzed by liquid chromatography, followed by mass spectrometry analysis (Figure 5B). The upper band (phosphorylated FLCN) was significantly enriched in peptides phosphorylated at Serines 62 and 73 (S62 and S73). FLCN phosphorylation on serine 62 was previously reported [17], [18], but these phosphorylation sites have never been associated with changes in cell cycle progression.

To confirm that phosphorylation on serine 62 and serine 73 is responsible for the shift in the FLCN banding pattern during G2/M transition, we engineered a phosphomimetic FLCN mutant (FLCN S62/73E) and a phosphoinactivating mutant (FLCN S62/73A). Both phosphomutants (FLCN S62/73E or FLCN S62/73A) were stably expressed in the FLCN-deficient UOK257 cells and were detected in both the cytosol and the nucleus, similarly to WT FLCN (Figure 5C). The FLCN serine to alanine mutant that is unable to undergo phosphorylation on Serines 62 and 73 (FLCN S62/73A) did not shift upwards in nocodazole arrested cells, while the phosphomimetic serine to glutamic acid mutant (FLCN S62/73E) constitutively migrated more slowly than WT FLCN and its migration (banding pattern) was not affected by nocodazole treatment (Figure 5D). Interestingly, the in-frame, tumor-associated FLCN ΔF157 mutant, was also phosphorylated during mitosis, although to a lesser degree than the FLCN WT protein (Figure 5D). In order to determine if phosphorylation of FLCN on S62 and S73 affects stability of the protein, we grew U20S cells either asynchronously or arrested at G2/M and inhibited *de novo* protein synthesis with cycloheximide (Figure 5E). Endogenous FLCN is a relatively stable protein with a half-life of approximately 6–8 hours (Figure 5E). The intensity of the phosphorylated form of the FLCN protein (upper band in nocodazole arrested cells) diminished prior to the dephosphorylated form (lower band) at the 4–6 hours time points, suggesting that FLCN S62/S73 phosphorylation has reduced stability (Figure 5E).

**Figure 6:**
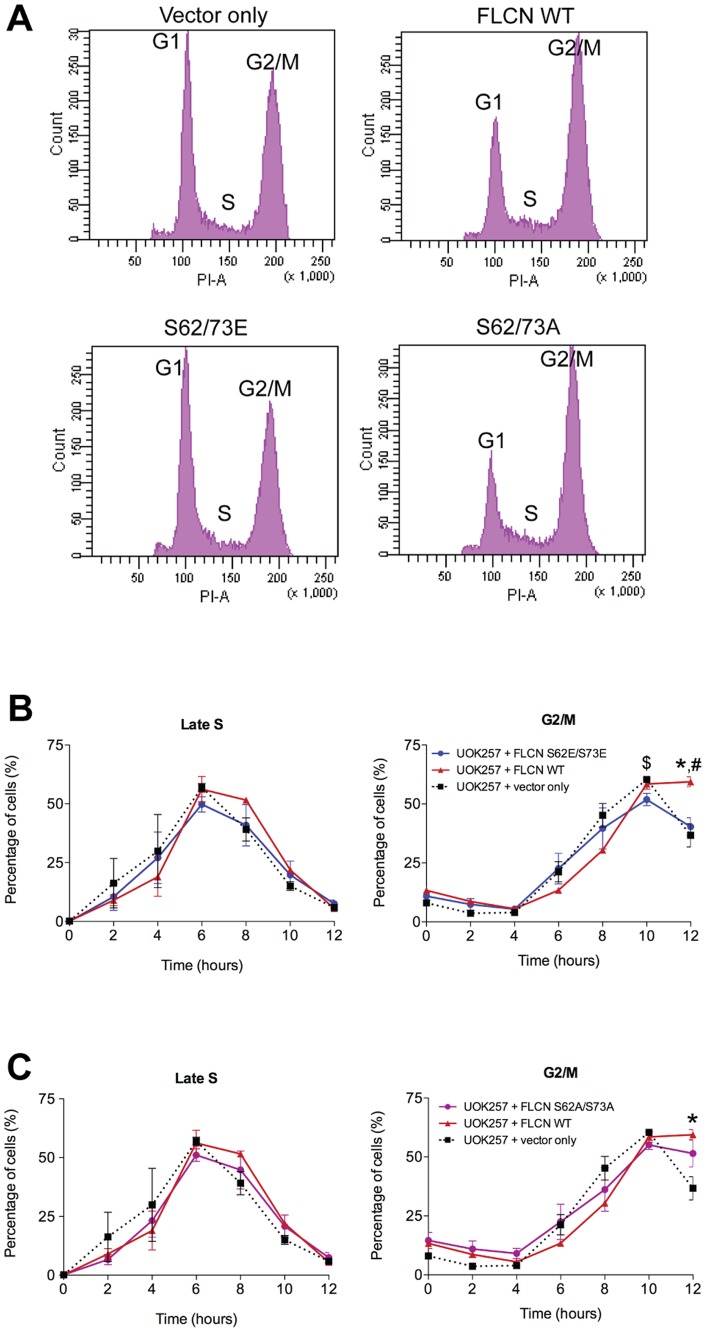
FLCN phosphomimetic mutations inactivate the effect of WT FLCN on cell cycle. *(A) A FLCN phosphomimetic mutant (S62/73E) results in more rapid progression through the cell cycle compared to the FLCN WT, while the FLCN phosphoinactivating mutant (S62/73A) retains the ability to delay cell cycle progression.* Representative cell cycle profiles from UOK257 cells and the isogenic UOK257 cells expressing FLCN WT, or the phosphomutants S62/73E or S62/73A, collected 12 hours after release from thymidine. UOK257 cells expressing FLCN WT or S62/73A have more cells in G2/M and fewer cells entering G1 compared to the FLCN null (vector only) and the S62/73E cells as measured by PI staining. (B) UOK257 (black line) and their isogenic counterparts reconstituted with WT FLCN (red line) or FLCN S62/73E mutant (blue line) were synchronized by double thymidine block. Cell cycle progression after release from thymidine was analyzed by FACS. Vertical axis indicates the percent of all cells registering in the specific phase of cell cycle. Horizontal axis indicates hours post thymidine release. The average of three experiments is presented (n = 3) in each panel; bars correspond to standard error of the mean (SEM). (C) Same as in (B), except that the reconstituted phosphomutant is FLCN S62/73A (purple line). For both (B) and (C), * indicates a statistically significant difference between UOK257+ vector only compared to UOK257+ FLCN WT at the corresponding time point; # indicates a difference between UOK257 FLCN WT and the UOK257+ phosphomutant (S62/73E or S62/73A); $ indicates a difference between UOK257+ vector only and UOK257+ phosphomutant (S62/73E or S62/73A) (One-way ANOVA, Tukey's post-test, p<0.05).

Lastly, we tested the ability of the phosphomutants (FLCN S62/73E or FLCN S62/73A) to delay cell cycle progression through the late S and G2/M phases. Phosphomimetic mutations (FLCN S62/73E) fail to delay cell cycle progression and exhibited a similar cell cycle profile as the FLCN deficient vector only controls (Figure 6A and B). This effect was most pronounced and reached statistical significance at the 12-hour time point of G2/M (Figure 6B). In contrast, the phosphoinactivating mutations (FLCN S62/73A) partially delayed progression through the cell cycle, similarly to the cells expressing WT FLCN (Figure 6A and C). These data suggest that orderly phosphorylation of FLCN occurs during the G2/M phases of the cell cycle and is required for the normal cellular functions of FLCN. In addition, our results demonstrate that, with regards to cell cycle, phosphorylation of FLCN on serines 62 and 73 results in an inactive form of the protein.

## Discussion

The FLCN gene encodes for a tumor suppressor phosphoprotein that localizes to the nucleus and the cytoplasm, but the biochemical mechanisms of FLCN function are currently unknown. In keeping with the physiology of many tumor suppressor genes, we hypothesized that FLCN affects cell cycle progression. The data presented herein demonstrate that FLCN deficient UOK257 cells progress more rapidly through the cell cycle compared to cells expressing WT FLCN. The difference between FLCN deficient and reconstituted UOK257 cells is maximal during the late S and G2/M phases of cell cycle, but it is unclear whether this reflects a primary delay during the late S phase or if FLCN affects both of these phases independently. Mutations in the FLCN gene associated with kidney tumorigenesis fail to slow cell cycle progression, suggesting a link between cell cycle progression and the tumor suppressor function of FLCN.

FLCN function has been tested in classic tumor suppressor assays, such as soft agar colony formation and tumor growth in mouse xenograft assays [14], [15], [29]. Testing FLCN species (such as tumor-associated mutants and phosphomutants) in the cell cycle progression assay we present here is an additional, significantly faster method, to differentiate between putative mutant forms and polymorphisms. Although the majority of FLCN germline mutations predict for a truncated form of the protein, missense mutations (such as K508R) and polymorphisms have been reported [10], [30], [31], [32]. In addition, this rapid *in vitro* assay can provide a reporter assay for testing the functional significance of putative FLCN interacting proteins and therefore accelerate insight into the biochemical functions of FLCN.

FLCN is an evolutionarily conserved protein and knocking out the mouse ortholog in mouse kidneys resulted in hyperplasia and tumorigenesis [11], [12], [13]. Analysis of the renal hyperplasia and tumors in *Flcn* knockout mice revealed increased cellular proliferation, but the cell cycle changes underlying this phenotype were not addressed [11]. Increased proliferation rate may be due to shortening of any or all phases of the cell cycle, or more rapid exit from quiescence. Here we show specifically, in both the MEF *Flcn* ^–/–^ and the UOK257 cells, that WT FLCN delays progression of cell cycle through the late S and G2/M phases. The ability of FLCN to alter cell cycle progression in both human and mouse cells further suggests that the tumor suppressor activity of FLCN is, at least in part, mediated by its ability to affect cell cycle progression.

Similarly to the phosphorylation activity of other cell cycle regulating tumor suppressor proteins, such as Rb and p53 [33], [34], [35], we observed that FLCN phosphorylation changes during the cell cycle. Phosphorylation of FLCN increases as cells progress through the G2/M checkpoint and returns to basal levels (equivalent to asynchronous cells) after exit from mitosis. Our results demonstrate that orderly changes in FLCN phosphorylation at different stages of the cell cycle are linked to FLCN's ability to slow progression through the late S and G2/M stages of the cell cycle. The introduction of the phosphomimetic FLCN S62/73E mutant into FLCN-deficient cells produces a G2/M transition phenotype resembling the null (UOK257 vector only) cells. In contrast, the S62/73A phosphoinactivating mutant leads to an intermediate phenotype between wild type and null. These differences are consistent, highly reproducible and telling of a biological phenomenon, namely the requirement of orderly phosphorylation followed by dephosphorylation to fulfill the wild type function. Phosphomimetic mutants (StoE) are inactive with regards to G2/M transition. Consistent with this hypothesis is the observation that the hyperphosphorylated form has a shorter protein half-life. The StoA mutant is partially active (presumably during early/mid S) but possibly fails to execute wild type functions related to G2/M. Since cells reconstituted with the FLCN phosphomutants displayed similar expression levels and cellular localization patterns as those reconstituted with WT FLCN, it is likely that phosphorylation of FLCN directly affects its ability to interact with partner proteins and activate signaling pathways. These interactions may occur in either the nuclear and/or cytoplasmic compartments of the cell.

In summary, these data demonstrate a novel function of the tumor suppressor protein FLCN on cell cycle progression and pinpoints this effect to the late S and G2/M phases. The biologic significance of this observation is supported by the findings that cells expressing tumor-associated FLCN mutants fail to exhibit a delay cell cycle progression. In addition, we show that the phosphorylation of FLCN changes during the cell cycle, and is likely responsible for FLCN's role in cell cycle regulation.

## Aknowledgments
